# Assessment of Environmental Enteric Dysfunction in the SHINE Trial: Methods and Challenges

**DOI:** 10.1093/cid/civ848

**Published:** 2015-11-11

**Authors:** Andrew J. Prendergast, Jean H. Humphrey, Kuda Mutasa, Florence D. Majo, Sandra Rukobo, Margaret Govha, Mduduzi N. N. Mbuya, Lawrence H. Moulton, Rebecca J. Stoltzfus

**Affiliations:** 1 Zvitambo Institute for Maternal and Child Health Research, Harare, Zimbabwe; 2 Blizard Institute, Queen Mary University of London, United Kingdom; 3 Department of International Health, Johns Hopkins Bloomberg School of Public Health, Baltimore, Maryland; 4 Division of Nutritional Sciences, Cornell University, Ithaca, New York

**Keywords:** infants, stunting, environmental enteric dysfunction, inflammation, IGF-1

## Abstract

Environmental enteric dysfunction (EED) is a virtually ubiquitous, but poorly defined, disorder of the small intestine among people living in conditions of poverty, which begins early in infancy and persists. EED is characterized by altered gut structure and function, leading to reduced absorptive surface area and impaired intestinal barrier function. It is hypothesized that recurrent exposure to fecal pathogens and changes in the composition of the intestinal microbiota initiate this process, which leads to a self-perpetuating cycle of pathology. We view EED as a primary gut disorder that drives chronic systemic inflammation, leading to growth hormone resistance and impaired linear growth. There is currently no accepted case definition or gold-standard biomarker of EED, making field studies challenging. The Sanitation Hygiene Infant Nutrition Efficacy (SHINE) trial in Zimbabwe is evaluating the independent and combined effects of a package of infant feeding and/or water, sanitation, and hygiene interventions on stunting and anemia. SHINE therefore provides an opportunity to longitudinally evaluate EED in a well-characterized cohort of infants, using a panel of biomarkers along the hypothesized causal pathway. Our aims are to describe the evolution of EED during infancy, ascertain its contribution to stunting, and investigate the impact of the randomized interventions on the EED pathway. In this article, we describe current concepts of EED, challenges in defining the condition, and our approach to evaluating EED in the SHINE trial.

A key hypothesis of the Sanitation Hygiene Infant Nutrition Efficacy (SHINE) trial is that environmental enteric dysfunction (EED), a subclinical disorder of the small intestine, is a major cause of child stunting [1]. Within SHINE, the cluster-randomized design will enable us to investigate the impact of water, sanitation, and hygiene (WASH) and infant and young child feeding interventions on the pathogenesis of EED, and to evaluate other causes and consequences of EED through observational substudies [1]. In this article, we discuss current concepts of EED, challenges in defining the condition, emerging biomarkers, and our approach to evaluating the EED pathway in the SHINE trial.

## CURRENT CONCEPTS OF EED

**Figure 1.**
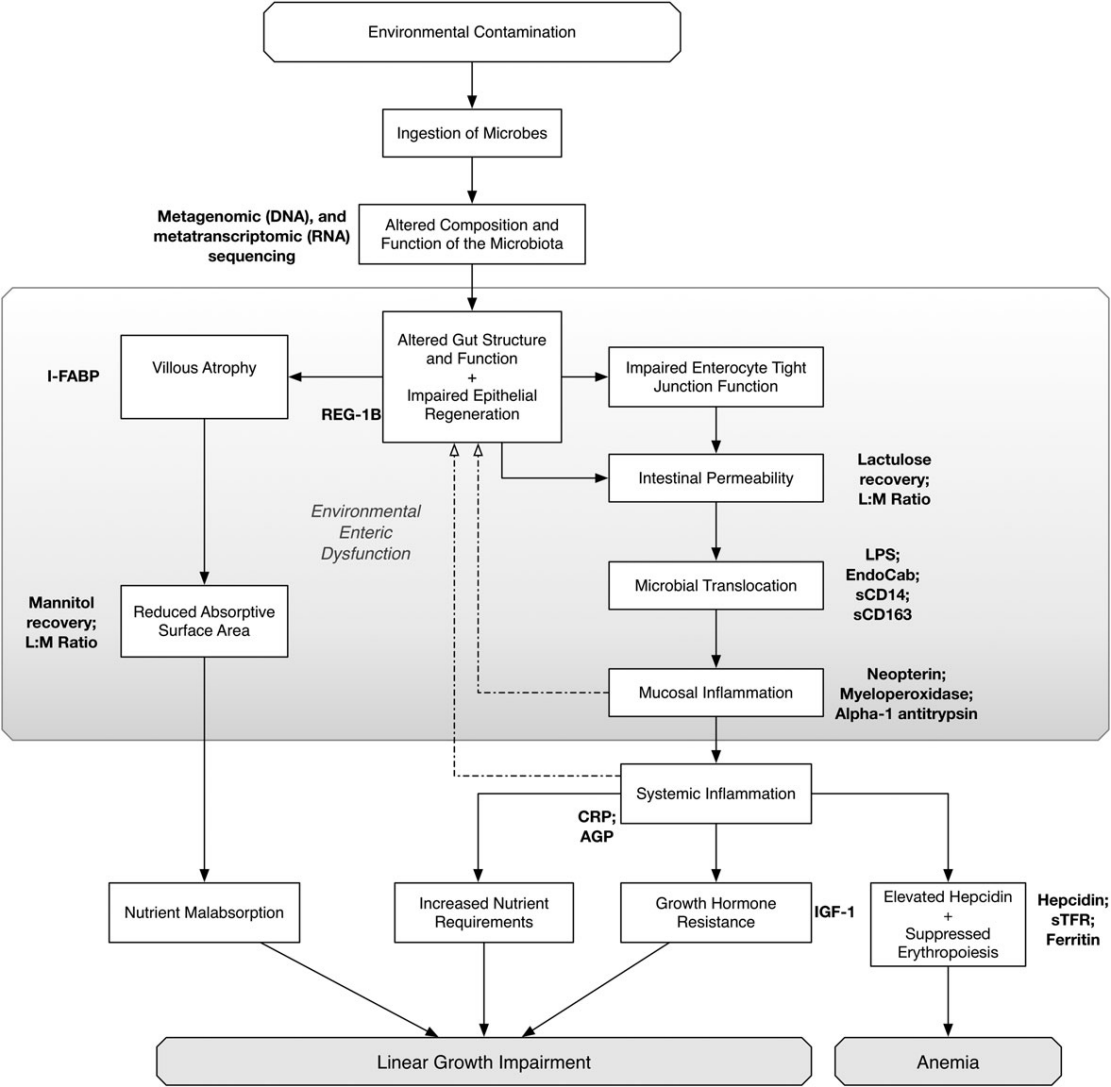
Hypothesized causal pathway to stunting through environmental enteric dysfunction. Abbreviations: AGP, α-1 acid glycoprotein; CRP, C-reactive protein; I-FABP, intestinal fatty acid binding protein; IGF-1, insulin-like growth factor 1; L:M, lactulose mannitol ratio; LPS, lipopolysaccharide; REG-1B, regenerating gene 1B; sCD14, soluble CD14; sCD163, soluble CD163; sTFR, soluble transferrin receptor.

EED represents a population-wide shift in gut structure and function in areas of poverty, where apparently healthy people have abnormal small intestinal biopsy findings, characterized by reduced villus height, increased crypt depth, and lymphocytic infiltration [2–5]. Villi become fused and broad, causing formation of leaves and ridges, which reduces the absorptive surface area of the gut and causes modest maldigestion and malabsorption of nutrients [6]. Dysregulation of tight junction proteins leads to increased small intestinal permeability, enabling microbial translocation and systemic inflammation [2, 7, 8]. We hypothesize that recurrent exposure to fecal pathogens and changes in the composition of the intestinal microbiota initiate this process, which leads to a self-perpetuating cycle of pathology (Figure 1), although the precise sequence of events remains unclear [9].

Whether EED is one condition, or has several distinct phenotypes, is unknown. There are multiple causes of intestinal damage in developing countries, which overlap and interact; the small intestine has a relatively limited repertoire of responses to insult, so several different exposures could mediate similar pathological findings [6]. EED appears almost universal in settings where WASH coverage and practices are suboptimal, enteric pathogen carriage is high, nutritional deficiencies are widespread, and other exposures (eg, mycotoxin contamination of staple foods) are common; however, the relative contribution of each is unknown [2, 6].

We lack understanding of the geographical variation in EED because of challenges in defining the condition consistently, although recent findings from the Malnutrition and Enteric Disease (MAL-ED) study, which used standardized methods to assess gut biomarkers in 8 birth cohorts across 3 continents, confirm that this condition is almost ubiquitous among young children in impoverished communities [10]. Infants across these diverse settings had elevated markers of intestinal inflammation that far exceeded levels typically seen among infants in developed countries [10]. We recently showed that Zimbabwean infants have extremely high plasma concentrations of intestinal fatty acid binding protein (I-FABP), indicating extensive damage to small-intestinal villi, with levels exceeding those reported among healthy children in Europe or among unhealthy children with celiac disease [11]. Furthermore, we found that infants with poor linear growth had elevated acute phase proteins and reduced concentrations of insulin-like growth factor 1 (IGF-1), suggesting a central role for chronic inflammation and growth hormone resistance in stunting [11].

We hypothesize that EED may have other adverse effects beyond linear growth failure. Induction of immune responses following oral vaccination may be impaired in the setting of an inflamed gut, contributing to reduced oral vaccine efficacy in developing countries [12]. The state of chronic inflammation that arises from EED may plausibly contribute to anemia, through elevation of hepcidin and/or anemia of inflammation, and to dysregulated immune ontogeny, leading to immunosenescence, or premature aging of the immune system. As the function of the microbiota-gut-brain axis becomes more apparent [13], it is plausible that EED contributes to impaired neurocognitive development, which is one of the most pervasive sequelae of the stunting syndrome [14]. Finally, we hypothesize that EED in women of reproductive age leads to microbial translocation and chronic inflammation during pregnancy, which may mediate adverse birth outcomes such as fetal growth restriction and prematurity, as has been shown for infection or inflammation at other extrauterine sites [15].

## CHALLENGES IN DEFINING EED

EED was first termed *tropical enteropathy* [3], but was renamed *environmental enteropathy* when it became clear that impoverished living conditions, rather than geographical location per se, were the most important determinants of this subclinical pathology [16]. A recent working group proposed the term *environmental enteric dysfunction* to better capture the functional, as well as structural, abnormalities associated with this condition [17]; however, the disorder remains poorly characterized [9]. A major obstacle to better understanding EED is that small-intestinal biopsies from young children are technically and ethically difficult to obtain, meaning that most studies now rely on noninvasive markers to define the disorder. Where small-intestinal biopsies have been examined in adults, the morphometric changes of EED can be quantified, but they do not appear to correlate well with functional biomarkers [2]. There is therefore no currently accepted case definition of EED, which provides a major problem for research studies [17].

## DEFINING EED IN THE SHINE TRIAL

The SHINE trial provides an opportunity to investigate the pathophysiology of the stunting syndrome, using the randomized interventions as a probe to explore mechanistic pathways [1]. We view EED as a primary gut disorder that drives a chronic systemic inflammatory process leading to growth hormone resistance, which limits growth (Figure 1) [18]. There is no gold-standard biomarker of EED, and it is unlikely that a single marker would ever define this condition. In agreement with others [17, 19], we consider that a range of biomarkers along this pathway will provide the most informative picture of the associations between gut pathology and linear growth (Figure 1, Table 1). It has been proposed that, in the absence of a reliable biomarker, an “enteropathy index” may be required, which would combine clinical and laboratory data to define EED [20].

**Table 1.** Biomarkers of Environmental Enteric Dysfunction

Domain	Biomarker	Method	Sample Type
Intestinal absorption	Mannitol recovery^a^	Mass spectrometry	Urine (2 h collection)
Intestinal inflammation	α-1 antitrypsin, neopterin, myeloperoxidase	ELISA	Stool
Enterocyte damage	I-FABP	ELISA	Plasma
Intestinal regeneration	REG-1B	ELISA	Stool
Intestinal barrier function	Lactulose recovery^a^	Mass spectrometry	Urine (2 h collection)
Microbial translocation	EndoCAb, LPS^b^, sCD14, sCD163	ELISA	Plasma
Systemic inflammation	CRP, AGP	ELISA	Plasma
Growth hormone activity	IGF-1	ELISA	Plasma

In a subgroup of infants recruited to SHINE (250 human immunodeficiency virus [HIV]–unexposed infants per trial arm, and all HIV-exposed infants), urine, stool, blood, and saliva samples are collected at 3, 6, 12, and 18 months of age. At 1 month of age, paired maternal and infant stool and blood are collected.

Abbreviations: AGP, α-1 acid glycoprotein; CRP, C-reactive protein; ELISA, enzyme-linked immunosorbent assay; EndoCAb, endotoxin core antibody; I-FABP, intestinal fatty acid binding protein; LPS, lipopolysaccharide; REG-1B, regenerating gene 1B; sCD14, soluble CD14; sCD163, soluble CD163; SHINE, Sanitation Hygiene Infant Nutrition Efficacy.

^a^ Lactulose-mannitol (LM) testing is conducted at 3, 6, 12, and 18 months. Prior to testing, a pre-LM urine sample is collected to measure baseline mannitol. Infants are fasted for at least 30 minutes before ingesting 2 mL/kg of a solution containing mannitol (50 mg/mL) and lactulose (250 mg/mL). Total urine is collected in an adhesive bag for 2 hours, during which time the mother is encouraged to feed her infant regularly to permit collection of an adequate volume of urine. Collected urine is preserved using chlorhexidine to prevent overgrowth of bacteria, measured, and taken back to the laboratory for storage at −80°C for subsequent measurement of lactulose and mannitol concentrations by mass spectrometry.

^b^ LPS will be measured in mothers only, because endotoxin-free conditions of blood collection cannot be guaranteed in infants.

## EED SUBSTUDY POPULATION

We have adopted a longitudinal approach to sample collection in a subgroup of infants enrolled to SHINE, which will enable us to describe the evolution of EED during infancy, ascertain its contribution to stunting, and investigate the impact of the randomized interventions on the EED pathway. All mothers reaching their 32-week gestational visit from 1 May 2014 through the end of the trial are invited to enroll in the EED substudy, for a total sample size of at least 1000 human immunodeficiency virus (HIV)–unexposed infants (250 per trial arm). HIV-unexposed infants are defined as those born to women testing HIV negative at baseline and/or 32 gestational weeks. All consenting HIV-infected mothers are enrolled, with infant analyses stratified by maternal HIV status, because of the likely impact of HIV exposure and infant cotrimoxazole prophylaxis on underlying causal pathways. Infants of women who seroconvert during follow-up (ie, women testing HIV negative at baseline and/or 32 weeks, but HIV positive at 18 months postpartum) will be excluded from analysis because of uncertain duration of HIV exposure. Specimens are collected from mothers and infants at 1 month of age (blood and stool), and from infants at 3, 6, 12, and 18 months (blood, stool, saliva, and urine).

## SPECIMEN COLLECTION, TRANSPORT, AND PROCESSING

Infant blood is collected at each time-point into a PAXgene tube (PreAnalytiX GmbH, Switzerland) for subsequent transcriptomic analysis, and an EDTA tube (BD Biosciences) for point-of-care hemoglobin measurement (HemoCue Hb 301, Angelholm, Sweden), CD4 count and HIV DNA polymerase chain reaction testing (HIV-exposed infants only), plasma storage (for biomarker analysis), and peripheral blood mononuclear cell (PBMC) isolation (for flow cytometry). Salivary samples are collected using oral swabs (Salimetrics LLC, Carlsbad, California); stool samples are collected into plain tubes without fixative, prior to ingestion of lactulose-mannitol (LM) solution, and urine is collected prior to and for 2 hours after administration of LM solution (see below). Samples are carried in cool boxes to field laboratories, where they are processed and aliquoted for storage at −20°C or −80°C with a backup generator.

## BIOMARKERS OF EED

Our choice of biomarkers (Table 1) is based on a combination of prior EED studies, extrapolation from other intestinal diseases, and biological plausibility, although the final panel of markers may change depending on emerging data. A consortium supported by the Bill & Melinda Gates Foundation is evaluating a number of candidate biomarkers, and in some sites work is under way to correlate biomarkers with gut biopsies or confocal laser endomicroscopic findings.

### Intestinal Markers

Stool samples will be used to measure intestinal inflammation and epithelial regeneration. The Mal-ED study showed that a combination of 3 fecal inflammatory markers predicted subsequent deficits in linear growth better than any single marker; we are therefore measuring fecal neopterin (GenWay Biotech, San Diego, California), myeloperoxidase (Immundiagnostik, Bensheim, Germany) and α-1 antitrypsin (Biovendor, Brno, Czech Republic) by enzyme-linked immunosorbent assay (ELISA), as previously described [10]. Regenerating gene 1β (REG-1B) protein is a C-type lectin family member, which can be measured in stool by ELISA (Techlab Inc, Blacksburg, Virginia) and reflects epithelial injury and regeneration [21]. In infants from Peru and Bangladesh, higher stool REG-1B concentrations at 3 months of age were independently associated with lower attained linear growth through 24 months [22].

The LM test remains widely used to assess intestinal absorptive capacity and permeability [20]. Among studies reporting LM ratios (or separate mannitol and lactulose excretion values) and growth in children, most [7, 23–26], but not all [27, 28], found inverse associations with linear growth. However, the test has certain limitations: first, it is a cumbersome test that requires fasting and prolonged urine collection from young infants; second, comparison across studies is difficult due to methodological differences in test performance and analysis [20]; third, the ingested solute load may alter gut transit times [29] and intestinal permeability [30, 31]; and fourth, analysis is expensive and technically challenging. Nevertheless, it remains a potentially useful marker in large field trials where biopsy samples are not feasible [20]. We are conducting LM tests on EED substudy infants in their homestead, using a standard operating procedure based on experience from the Mal-ED study [32]. Although 5-hour urine collections have been used in many previous studies that included LM tests, longer durations are more demanding on caregiver and fieldworker time, and lactulose recovery partly reflects colonic permeability [33]. Shorter collection times have a practical advantage for fieldworkers, and lactulose recovery better reflects small intestinal permeability [33]; we have therefore chosen a 2-hour urine collection procedure in SHINE. The disadvantages of a 2-hour collection are that the test sometimes has to be extended in duration until the infant passes urine, and analysis must be by mass spectrometry (rather than high-performance liquid chromatography) because of lower urinary sugar concentrations (Margaret Kosek, personal communication). Because mannitol can be found naturally in the urine [30], a baseline urine sample is collected where possible prior to dual sugar ingestion to correct for this in analysis; however, the need to collect and analyze 2 samples per child increases test complexity and costs further.

A dedicated facility in the central Harare laboratory manufactures 20-mL aliquots of LM solution (containing 250 mg/mL lactulose and 50 mg/mL mannitol in sterile water). LM solution is maintained at 2°C–8°C and transported by cold chain to field laboratories, where it is carried in a dedicated cool box to the participant's home. Upon arrival, the fieldworker weighs the infant and attaches an adhesive urine bag; the mother continues to breastfeed the infant until a baseline urine sample is collected. The infant then fasts for 30 minutes prior to administration of 2 mL/kg LM solution by oral syringe or cup. A new urine bag is attached and the infant fasts for 30 minutes prior to resuming breastfeeding. The bag is emptied frequently over a 2-hour period, and each aliquot of urine is preserved with chlorhexidine to prevent microbial overgrowth, then placed in a cool box for storage. Two hours after LM ingestion, the test is stopped; if an infant has not passed any urine, the test is extended until urine is collected. The total volume of LM urine is measured in the field laboratory and samples are frozen in aliquots for subsequent measurement of lactulose and mannitol by mass spectrometry.

Plasma I-FABP is easy to measure using a commercial ELISA (Hycult Biotech, Uden, the Netherlands), reflects small-intestinal villus damage, and has a short half-life, meaning it is a dynamic marker of the intestinal epithelium [34]; it is a useful biomarker of celiac disease [35], and studies are under way in Zambia to correlate I-FABP concentrations with epithelial breaches detected by confocal laser endomicroscopy (Paul Kelly, personal communication).

### Microbial Translocation and Inflammation

It is hypothesized that impaired intestinal barrier function enables organisms and microbial products to translocate from the gut to the mesenteric lymph nodes, liver, and systemic circulation [17–19]; however, this is a difficult domain of the causal pathway to evaluate. Lipopolysaccharide (LPS, or endotoxin) is found in the outer membrane of gram-negative bacteria, making it a plausible marker of translocation from the gut; however, measurement of plasma LPS requires blood specimens to be collected under scrupulously endotoxin-free conditions, and this is challenging in young infants, in whom a closed venipuncture system often cannot be used. LPS elicits a strong immune response, and several studies have measured immunoglobulin M or immunoglobulin G antibodies to the core domain of endotoxin (EndoCAb) by ELISA, finding variable associations with growth [7, 11, 36]; there are also technical problems with the commercially available assay [32]. LPS also stimulates circulating monocytes and tissue macrophages to release soluble CD14 and soluble CD163; while these may plausibly represent alternative markers of translocation, no studies to date have demonstrated relationships with linear growth.

It is postulated that translocated microbial products stimulate innate immune cells to release proinflammatory cytokines (interleukin [IL] 6, IL-1β, and tumor necrosis factor α), which then trigger hepatic synthesis of acute phase proteins, such as C-reactive protein (CRP) and α1-acid glycoprotein (AGP) [17–19]. In a previous study, we showed that a range of inflammatory markers (IL-6, CRP, AGP) were inversely associated with IGF-1 concentrations; the average level of CRP between 6 weeks and 12 months of age had the strongest associations with stunting [11]. CRP is easy to measure on small quantities of plasma using a commercial assay (R&D Systems, Inc, Minneapolis, Minnesota), making it appealing for infant studies; AGP is similarly straightforward to measure by ELISA (R&D Systems, Inc), and is an acute phase protein with a longer half-life [37]; however, a broader range of multiplexed pro- and anti-inflammatory cytokines may allow a more detailed evaluation of the inflammatory milieu. In a subgroup of infants, we will undertake immunophenotyping on thawed PBMCs by flow cytometry, to describe lymphocyte ontogeny, activation, and senescence across trial arms.

### Growth Hormone–IGF-1 Axis

Growth hormone is released in a pulsatile manner and stimulates hepatic synthesis of IGF-1, which circulates in a ternary complex with its principal binding protein, IGF binding protein 3, and an acid-labile subunit [38]. IGF-1 stimulates clonal expansion of chondrocytes to directly promote linear growth, and is therefore an attractive biomarker in studies of stunting as it directly mediates the effects of growth hormone [39]. Plasma IGF-1 is easily measured by ELISA (R&D Systems, Inc), and we have shown that concentrations are significantly reduced in infants with poor linear growth [11], likely due to growth hormone resistance in the setting of chronic inflammation [40].

### Oral Vaccine Responses

In a subgroup of infants, we will measure plasma immunoglobulin A (IgA) responses to rotavirus and poliovirus, prevaccination (1 month) and postvaccination (3 months), to compare seroconversion rates and IgA titers between infants. We will investigate whether vaccine immunogenicity is related to biomarkers of EED; whether WASH interventions can augment vaccine immunogenicity; and whether infants at risk of oral vaccine failure can be identified prior to vaccination.

### Biomarkers of Anemia

In addition to the biomarkers of EED already described, we will measure a panel of markers (ferritin, soluble transferrin receptor, and hepcidin) to better understand the relative contributions of inflammation and iron deficiency to infant anemia.

### Morbidity Data

Mothers of infants in the EED substudy will keep a daily morbidity diary, using stickers to record episodes of illness (diarrhea; blood or mucus in stools; cough; fast or difficult breathing; fever; or lethargy preventing feeding) so that incidence and prevalence of acute and persistent diarrhea, and other intercurrent infections, can be estimated.

## ANALYSIS APPROACH

To test hypotheses pertaining to EED, an intent-to-treat analysis will be carried out on each domain in the causal pathway. First, we will use generalized estimating equations to construct prediction equations for child length-for-age *z* score (LAZ) at 18 months. We will multiply regression coefficient estimates for the treatment effect of the WASH intervention on EED exposure among children in the WASH arm of the trial by those partial regression coefficients for the association of EED with child LAZ among children in the standard-of-care arm to attain the estimated effect of the WASH intervention on child LAZ at 18 months as mediated by EED. Variability in each domain of the pathway will be characterized either as a continuous function or as categories. For some domains, we will explore the computation of summary indices, such as the disease activity score developed by Mal-ED using 3 intestinal inflammatory markers [10], which has been adopted by other groups [41], or an “enteropathy index” [20]. Thereafter, for each link in the pathway, we will study the association between variability in the upstream domains/variables and variability in the subsequent domains/variables. Second, we will calculate maximum likelihood estimates of these same parameters using a path analysis approach. Path analysis techniques enable a more rigorous assessment of mediation than is possible through causal step approaches [42]. We will estimate the indirect effect of the WASH intervention on child LAZ as mediated by EED. The same approach will be taken to assess mediation by diarrhea, to evaluate the relative contributions of diarrhea and EED in the pathogenesis of stunting.

## CONCLUSIONS

EED is a virtually ubiquitous, but poorly defined, disorder of the small intestine of people living in conditions of poverty that begins early in infancy and persists. It may plausibly impact linear growth, neurodevelopment, oral vaccine responses, and immune ontogeny, and several trials are under way to evaluate the impact of preventive or treatment approaches for EED [43]. Several research groups are actively evaluating novel markers of EED, but currently there is no accepted case definition or gold-standard biomarker, making field studies challenging. The SHINE trial provides an opportunity to longitudinally explore disease mechanisms, using the most robust current and emerging biomarkers of EED to better understand the impact of public health interventions on the causal pathway to stunting.
